# Sperm morphology and chromatin integrity in Swedish warmblood stallions and their relationship to pregnancy rates

**DOI:** 10.1186/1751-0147-50-2

**Published:** 2008-01-07

**Authors:** Jane M Morrell, Anders Johannisson, Anne-Marie Dalin, Linda Hammar, Thomas Sandebert, Heriberto Rodriguez-Martinez

**Affiliations:** 1 Division of Reproduction, Department of Clinical Sciences, Faculty of Veterinary Medicine and Animal Science, Swedish University of Agricultural Sciences (SLU), Ullsvägen 14C, Clinical Centre, Box 7054 Ultuna, SE-750 07 Uppsala, Sweden; 2 Department of Anatomy, Physiology and Biochemistry, Box 7011, Ultuna, SE-750 07 Uppsala, Sweden; 3 Flyinge AB, SE-240 32 Flyinge, Sweden

## Abstract

**Background:**

Artificial insemination is not as widely used in horses as in other domestic species, such as dairy cattle and pigs, partly because of the wide variation in sperm quality between stallion ejaculates and partly due to decreased fertility following the use of cooled transported spermatozoa. Furthermore, predictive tests for sperm fertilising ability are lacking. The objective of the present study was to assess sperm morphology and chromatin integrity in ejaculates obtained from 11 warmblood breeding stallions in Sweden, and to evaluate the relationship of these parameters to pregnancy rates to investigate the possibility of using these tests predictively.

**Methods:**

Aliquots from fortyone ejaculates, obtained as part of the normal semen collection schedule at the Swedish National Stud, were used for morphological analysis by light microscopy, whereas thirtyseven were used for chromatin analysis (SCSA) by flow cytometry. The outcome of inseminations using these ejaculates was made available later in the same year.

**Results:**

Ranges for the different parameters were as follows; normal morphology, 27–79.5%; DNA-fragmentation index (DFI), 4.8–19.0%; standard deviation of DNA fragmentation index (SD_DFI) 41.5–98.9, and mean of DNA fragmentation index (mean_DFI), 267.7–319.5. There was considerable variation among stallions, which was statistically significant for all these parameters except for mean_DFI (*P *< 0.001, *P *< 0.01, *P *< 0.001 and *P *< 0.2 respectively). There was a negative relationship between normal morphology and DFI (P < 0.05), between normal morphology and SD_DFI (*P *< 0.001), and between normal morphology and mean_DFI (P < 0.05). For specific defects, there was a direct relationship between the incidence of pear-shaped sperm heads and DFI (*P *< 0.05), and also nuclear pouches and DFI (*P *< 0.001), indicating that either morphological analysis or chromatin analysis was able to identify abnormalities in spermiogenesis that could compromise DNA-integrity. A positive relationship was found between normal morphology and pregnancy rate following insemination (r = 0.789; *P *< 0.01) and a negative relationship existed between DFI and pregnancy rate (r = -0.63; *P *< 0.05). Sperm motility, assessed subjectively, was not related to conception rate.

**Conclusion:**

Either or both of the parameters, sperm morphology and sperm chromatin integrity, seem to be useful in predicting the fertilising ability of stallion ejaculates, particularly in determining cases of sub-fertility.

## Background

The number of artificial inseminations (AI) carried out in mares has increased considerably over recent years but, at around one million inseminations worldwide annually, use of this technique lags far behind that seen in other domestic animals, particularly those species used for food production. More than 100 million inseminations in cattle and more than 250 million inseminations in turkeys are carried out annually. Some of the limiting factors in the use of AI for mares are believed to be as follows: (i) highly variable quality between stallions and between ejaculates [1]; (ii) a decline in conception rate associated with the increased use of cooled transported spermatozoa rather than AI with fresh spermatozoa immediately after semen collection; (iii) lack of suitable on-site tests for sperm quality assessment [2]. Variation in stallion semen quality has been attributed to the selection of breeding sires based only on competitive performance [1].

Sperm quality, being multi-factorial, is difficult to define, and the different factors vary in significance between different species. Currently most studs rely on a subjective assessment of sperm motility as the sole determinant of sperm quality in insemination doses, although its correlation with fertility tends to be low [3]. Recently it has been suggested that a battery of physical and functional *in vitro *tests should be carried out on spermatozoa in an attempt to predict their likely fertilising capacity [1,2]. Such tests include sperm morphology [3-5] and chromatin integrity [6]. Disturbances in spermatogenesis give rise to sperm morphological abnormalities, although a wide range of sperm abnormalities may be acceptable for normal stallions if the proportion of spermatozoa with normal morphology is above 50% [7,8]. Specific morphological abnormalities are related to male sub-fertility [4].

Studies with human *in vitro *fertilization (IVF) have identified chromatin integrity as being of considerable importance in determining the outcome of assisted reproduction. A study in fertile stallions, in which the Sperm Chromatin Structure Assay (SCSA) was used to evaluate sperm chromatin [6], revealed a negative correlation between fertility and chromatin damage in sperm doses for AI [9,10]. The SCSA assay uses acridine orange to stain the sperm DNA, which is then analysed by flow cytometry to evaluate the ratio of single-stranded (abnormal) and double-stranded (normal) DNA present in individual spermatozoa.

The objective of the study reported here was to investigate whether sperm morphology and chromatin integrity can be used predictively in evaluating the quality of stallion spermatozoa for AI, using aliquots of ejaculates collected from stallions on a commercial stud in Sweden.

## Methods

### Animals and husbandry

Warmblood stallions of breeding age (7 to 23 years old) were housed under standard husbandry conditions at Flyinge AB, Flyinge, Sweden. Semen was collected up to three times a week during the normal breeding season (April to August in Sweden) as part of a commercial enterprise. Ethical permission for the study was granted by the Ethics Committee on Animal Experimentation, Uppsala, Sweden (C107/6): all experiments were carried out according to recognized international guidelines. The ejaculates used in this study were collected over a 15 day period in June 2006, with aliquots (6 mL) of the extended ejaculates being made available for the experiments. The remainder of the ejaculates was processed and supplied to customers for AI according to the usual procedures at the stud.

The ejaculates were extended 1:1 (v/v) with either Kenney's extender or Nørlund medium (see below) at 37°C. The Kenney's extender was used for stallions F, Y, T, A, R and K, while Nørlund medium was used for stallions H and W. Note: the stud personnel had recognized low pregnancy rates following AI with cooled, transported sperm doses for these two stallions from previous years, and were testing a different extender for these stallions in an attempt to rectify this situation.

### Media

Kenney's extender: to 100 mL distlled water were added glucose (4.9 g), skimmed milk powder (2.4 g), dihydrostreptomycin (0.15 g) and penicillin (sodium salt) (0.15 g).

Nørlund medium: an egg yolk-containing extender, purchased from Nørlunds Equine Hospital, Rue de Lund, 8653 Them, Denmark. (Note: The manufacturer is not prepared to reveal the ingredients contained in this extender).

### Sperm concentration

The concentration of spermatozoa in the original ejaculate was measured using a Spermacue photometer (Minitüb, Tiefenbach, Germany).

### Subjective estimation of motility

Subjective motility assessment was performed on aliquots (5.0 μL) of the extended ejaculate, using phase contrast light microscopy (x100) on a heated microscope stage (38°C), immediately after adding the extender and once daily until the motility had dropped to 20%. Sperm samples were stored at 4°C.

### Sperm Morphology

Smears of extended ejaculates were made on clean glass slides. In addition, a few drops of each sperm suspension were added to buffered formaldehyde [11]. Two hundred spermatozoa in these wet smears were evaluated by experienced laboratory assistants using phase contrast microscopy at 1,000× magnification with an immersion objective lens. Spermatozoa displaying morphological abnormalities (proximal cytoplasmic droplet, loose heads, abnormal acrosomes, nuclear pouches, abnormal mid-piece, simple coiled tails, tail coiled under the head, and double coiled tails) were identified. For sperm head morphology, 500 spermatozoa were examined in Williams-stained slides using a light microscope at 1,000× magnification, and the numbers of sperm head abnormalities (pear shape, narrow-based, abnormal contour, lack of development, narrowness, abaxial implantation, and abnormal loose heads) were identified. The staining method used was the modification of William's method [12] described by Lagerlöf [13]. All morphological abnormalities in the spermatozoa were recorded (i.e. both head and tail abnormalities may be present in one spermatozoa). The mean proportion of morphologically normal spermatozoa was estimated as the remaining proportion left from total abnormal spermatozoa counted both in wet smear and Williams stain (100 total abnormalities). Note: spermatozoa with a distal droplet but otherwise normal were counted as normal.

### Sperm chromatin structure assay

Abnormal chromatin structure was defined as the susceptibility of sperm DNA to undergo acid-induced denaturation *in situ*. Following exposure of the prepared DNA to acridine orange (AO), the degree of chromatin integrity was analysed by flow cytometric measurement (FCM) of the metachromatic shift from green (stable, double-stranded DNA) to red (denatured, single-stranded DNA) AO fluorescence [6]. This shift was expressed as the ratio of red to total (i.e. red and green) fluorescence intensity. In the SCSA, this ratio was calculated for each spermatozoon within a sample and the results were expressed as the ratio of single stranded to double stranded DNA [DFI (DNA fragmentation index, %), its standard deviation (SD_DFI) and the measure of single stranded DNA (mean_DFI)] [14].

In the present study, the procedure originally developed by Evenson et al. [6] and later described in detail by Januskauskas et al. [15,16] was followed. Aliquots of extended semen or centrifuged sperm preparations were mixed with TNE buffer (0.15 M NaCL, 0.01 M Tris-HCL, 1 mM EDTA (ethylenediaminetetra-acetic acid), pH 7.4) to a final sperm concentration of approximately 2 × 10^6 ^cells/mL. An aliquot of 0.2 mL was immediately frozen in liquid nitrogen vapour (LN_2_) and then transferred to an ultra-cold freezer (-80°C), where it was stored until further processing and FCM analysis. This procedure has been used for many years in our laboratory without any evidence of deterioration of the DNA samples during processing or storage. Samples of stallion spermatozoa with known high chromatin instability were processed in the same manner and served as controls for the procedure.

Just prior to analysis, the samples were thawed on crushed ice. The thawed, TNE-extended spermatozoa were subjected to partial DNA denaturation *in situ *(by mixing with 0.4 mL of a low pH detergent solution containing 0.17% Triton X-100, 0.15 M NaCl and 0.08 N HCl, pH 1.2), followed 30 seconds later by staining with 1.2 mL of AO (6 μg/ml in 0.1 M citric acid, 0.2 M Na_2_HPO_4, _1 mM EDTA, 0.15 M NaCL, pH 6.0). The stained samples were analysed within 3–5 minutes of AO staining. Samples of stallion spermatozoa with known high chromatin instability were processed in the same manner and served as controls for the procedure (Reference sample for SCSA): these reference samples were previously analysed samples of stallion spermatozoa found to have high (30–50%) DFI-values. When samples having such high DFI values are encountered, the remainder of the sample is aliquoted and re-frozen for subsequent use as a reference sample.

Measurements were made on a FACStar Plus flow cytometer (Becton Dickinson, San José, CA, USA) equipped with standard optics. Acridine orange was excited with an Ar (argon) ion laser (Innova 90; Coherent, Santa Clara, CA, USA) at 488 nm, running at 200 mW. As previously mentioned, in association with double-stranded DNA, AO fluoresces green (530± 30 nm, as detected using the FL 1 detector), but in the presence of single-stranded DNA the fluorescence is red (>630 nm, as detected with the FL 3 detector). The fluorescence stability of the flow cytometer was monitored daily using standard beads (Fluoresbrite plain YG 1.0 μM; Polysciences Inc., Warrington, PA, USA). Equivalent instrument settings were used for all samples. From each sample a total of 10,000 events were measured at a flow rate of ~200 cells/s. Data collection was carried out using CellQuest, version 3.3 (Becton Dickinson, San José, CA, USA). Further calculations were performed using FCS Express version 2 (De Novo Software, Thornhill, Ontario, Canada).

### Pregnancy rate

The mean pregnancy rate (PR) for the ejaculates used in this study was available later in the same year. For the purposes of this study, PR was taken to be:

PR = (no. mares pregnant/no. mares inseminated) × 100 (%)

Note: the stud where the stallions were kept was a commercial enterprise selling insemination doses to the owners of mares at various locations around Sweden. Therefore, no details of the insemination technique, oestrus and pregnancy detection are available.

### Statistics

Analysis of variance [17] was performed on mean values of the following parameters: the proportion of spermatozoa with normal morphology, DFI, SD_DFI and mean_DFI values for each stallion. Differences were considered to be statistically significant if *P *< 0.05. A regression analysis [17] was carried out on the following: normal morphology with PR; parameters of SCSA with PR, with the incidence of specific morphological defects, with sperm survival and with age of stallion. Again, values of *P *< 0.05 were considered significant.

## Results

The characteristics of the 41 ejaculates made available for this study are shown in Table 1. There was considerable variation among stallions in terms of sperm concentration and motility in the ejaculates: however, only sperm concentration between stallions differed significantly (*P *= 0.03). Differences in sperm concentration among ejaculates for each stallion were not significant.

**Table 1:** Quantitative and qualitative semen characteristics of stallion ejaculates (number of ejaculates per stallion within brackets) used in this study (mean ± SD).

**Stallion (no. ejaculates)**	**Concentration (×10^6^/mL)**	**Motility (%)**	**Spermatozoa with normal morphology (%)**
**A (4)**	201 ± 67	69 ± 13	69.9 ± 8.3
**F (4)**	356 ± 58	57 ± 14	75.6 ± 2.8
**G (3)**	216 ± 44	55 ± 8.7	77.5 ± 4.0
**H (3)**	228 ± 50	73 ± 5	31.2 ± 4.0
**K (4)**	162 ± 88	68 ± 5	71.8 ± 2.1
**Q (4)**	254 ± 88	71 ± 6	66.5 ± 7.8
**R (4)**	254 ± 86	65 ± 5	72.5 ± 2.8
**T (4)**	165 ± 47	68 ± 6	73.1 ± 2.1
**W (3)**	175 ± 38	63 ± 6	50.3 ± 2.9
**Y (4)**	276 ± 51	75 ± 6	77.4 ± 3.3
**Z (4)**	189 ± 16	73 ± 6	66.8 ± 3.6

Notes. Concentration was determined by Spermacue. Differences between stallions were significant for concentration (P = 0.03) and normal morphology (P < 0.001) but not for motility (P0 0.1). Differences between ejaculates for each stallion were not significant for concentration, motility or normal morphology.

The proportion of morphologically normal spermatozoa in the extended ejaculates varied between stallions (*P *< 0.001) (Table 1). The variation among stallions was highly significant but the variation between ejaculates (within stallions) was not significant. Furthermore, there was considerable variation between stallions in the prevalence of individual abnormalities although differences within stallions were not significantly different except for abnormal acrosomes (*P *< 0.05). Of the morphological abnormalities observed, the most prevalent in the extended semen were proximal cytoplasmic droplets (7–27%), pear-shaped heads (3.6–7%), heads with narrow bases (1.5–5%) and narrow heads (1–3%).

Regarding assessment of chromatin integrity (performed on 37 ejaculates), the mean (± SD) values for DFI, SD_DFI and mean_DFI for each of the 11 stallions, and the overall mean (± SD) for all ejaculates are shown in Table 2. Variation among stallions was significant for DFI (*P *< 0.01) and for SD_DFI (*P *< 0.001) but not for mean_DFI (*P *< 0.2). However, variation between ejaculates for individual stallions was significant only for mean_DFI (*P *< 0.05).

**Table 2:** Chromatin damage in stallion spermatozoa indicated by DFI (DNA fragmentation index, %), its standard deviation (SD_DFI) and the measure of single stranded DNA (mean_DFI). Values are expressed as means ± SD of aliquots of extended ejaculates from eleven stallions (n = 37).

**Stallion (no. ejaculates)**	**DFI**	**SD_DFI**	**Mean DFI**
**A (3)**	5.3 ± 0.5	47.7 ± 67.0	278.6 ± 9.4
**F (4)**	8.7 ± 3.3	48.6 ± 6.6	280.2 ± 16.2
**G (3)**	9.2 ± 1.4	48.6 ± 7.0	273.9 ± 3.2
**H (3)**	17.9 ± 4.5	64.0 ± 8.7	291.7 ± 13.1
**K (4)**	12.5 ± 2.8	58.7 ± 4.6	267.0 ± 6.5
**Q (3)**	5.7 ± 1.1	46.3 ± 2.8	277.8 ± 4.6
**R (3)**	8.4 ± 3.6	54.7 ± 6.4	283.6 ± 7.3
**T (3)**	11.1 ± 6.3	56.3 ± 5.3	287.5 ± 13.1
**W (3)**	13.9 ± 3.0	86.5 ± 13.4	302.5 ± 14.8
**Y (4)**	11.4 ± 2.7	56.7 ± 4.1	288.8 ± 4.1
**Z (4)**	12.6 ± 2.6	86.4 ± 8.1	297.8 ± 9.7
**Overall Mean ± SD**	11.6 ± 13.4	56.4 ± 14.4	311.0 ± 8.37
**Range**	4.8–19.0	41.5–98.9	267.7–319.5

Notes: DFI gives a measure of the proportion of spermatozoa with damaged chromatin; SD_DFI is the standard deviation of DFI; mean_DFI is a measure of single stranded DNA. Variation between stallions is statistically significant for DFI and SD_DFI (P < 0.001), variation between ejaculates is significant only for mean_DFI (P < 0.05).

Correlation analysis was performed for DFI, SD_DFI, and mean DFI and (i) normal morphology, (ii) total sperm head morphology, and (ii) the incidence of specific morphological defects. There was a negative relationship between normal morphology and DFI (r = -0.34, P <0.05), and between normal morphology and SD_DFI, (r = -0.43, *P *< 0.01). For specific abnormalities, there was a direct relationship between the incidence of pear-shaped heads and DFI (r = 0.34; *P *< 0.05), nuclear pouches and DFI (r = 0.47; *P *< 0.01), mid-piece defect and DFI (*P *< 0.01); between detached heads and SD_DFI (*P *< 0.01); and between detached heads and mean_DFI (*P *< 0.05). There was no statistically significant relationship between the other morphological defects and either DFI, SD_DFI or mean_DFI, nor between DFI and total sperm head abnormalities, nor between the age of stallion or length of sperm survival (defined for the purposes of this study as the number of days sperm motility was >20%) and the SCSA parameters.

**Figure 1:**
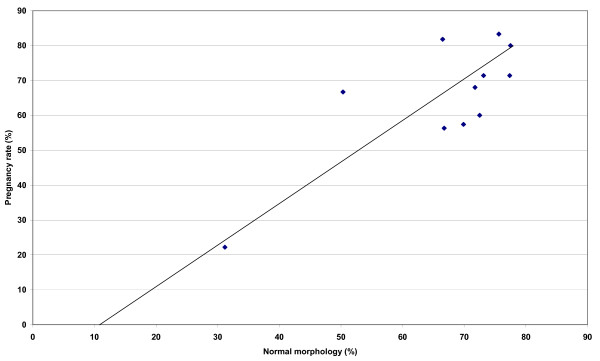
Relationship between normal morphology (%) and pregnancy rate (%) following artificial insemination with cooled, transported stallion spermatozoa derived from the ejaculates tested in this study (n = 41). The line of best fit for the data points is shown. Note: Statistics r = 0.789; P < 0.01.

**Figure 2:**
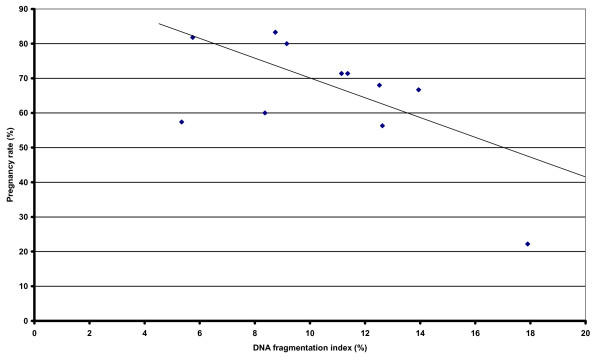
Relationship between DNA fragmentation rate (DFI) and pregnancy rate (%) following artificial insemination with cooled, transported stallion spermatozoa from the ejaculates tested in this study (n = 37). The line of best fit for the data points is shown. Note: Statistics r = -0.63; P < 0.05).

The mean PR of these insemination doses, obtained retrospectively, is shown in Table 3. There was a positive relationship between % normal morphology and PR following insemination (r = 0.789; *P *< 0.01) (Figure 1), and a negative relationship between DFI and PR (r = -0.63; P <0.05), with higher values of DFI being associated with a lower number of pregnancies following artificial insemination (Figure 2).

**Table 3:** Pregnancy rates (%) for the 41 ejaculates from 11 stallions used in this study, derived from the number of mares diagnosed pregnant divided by the number of inseminated mares.

**Stallion**	**No. mares pregnant/no. mares inseminated**	**Pregnancy rate (%)**
A	27/47	57.4
F	10/12	83.3
G	12/15	80.0
H	8/36	22.2
K	17/25	68.0
Q	9/11	81.8
R	3/5	60.0
T	10/14	71.4
W	2/3	66.7
Y	5/7	71.4
Z	27/48	56.3

## Discussion

Values for concentration and sperm motility were within the normal range for this species [18]. Considerable variation in these parameters between stallions has also been observed by others [1,19], although in this study there was no difference between ejaculates from each stallion collected over a short time period (15 days) at the height of the breeding season. Seasonal variation has been reported previously among stallion ejaculates [19].

The observations of a positive relationship between normal morphology and PR together with a negative relationship between DFI and PR are in accordance with other findings [4,9,10]. The relationship seen here between normal morphology and intact chromatin is also in accordance with previous observations. The link between the presence of certain morphological defects and chromatin damage is interesting and warrants further study, particularly those defects related to the condensation of the chromatin of the round spermatids during spermiogenesis. As such, there was a clear relationship between the presence of nuclear pouches and damaged chromatin. Therefore, the presence of pear-shaped heads and/or nuclear pouches in stallion spermatozoa could be useful as markers for suspected chromatin damage in this species, when the resources for the SCSA are lacking (for instance when no flow cytometer is available). A significant relationship between DFI and the occurrence of pear-shaped heads has been reported previously for boar spermatozoa, using the Sperm Chromatin Dispersion test (SCD) [20]. A strong relationship between the incidence of midpiece defects and damaged chromatin, as well as detached heads and SD_DFI, was observed in this study, although we do not have any logical, biological explanation for these relationships.

It is not unusual to encounter a number of abnormal sperm forms in any ejaculate; only when present in large numbers are morphological abnormalities associated with impaired fertility in the stallion [21]. The situation may be somewhat different in other species: for example, statistically significant correlations have been found between abnormal heads, nuclear pouches and proximal cytoplasmic droplets and fertility (56-day non-return rate) in bull ejaculates [22]. Bent tails and single coiled tails may be caused by derangement in the secretion of accessory fluids: bent tails may also result from pH and osmotic tension of the semen extender, while single coiled tails may be a preparation artifact. It has been suggested that causes for concern in the differential spermiogram would include less than 30% morphologically normal spermatozoa, more than 10% immature germ cells, more than 30% abnormal sperm heads and/or mid-piece defects, or more than 25% spermatozoa with proximal cytoplasmic droplets [4]. Such levels are usually associated with decreased fertility.

One of the stallions in the study reported here, stallion H, had a mean of only 31.2% morphologically normal spermatozoa in the three ejaculates examined, very close to the 30% cut-off point mentioned above [4]; of the abnormalities observed, 25% were midpiece defects. Insemination of the three ejaculates included in this study produced only two pregnancies in 13 mares, well below the pregnancy rates of ejaculates from the remaining stallions. Another stallion (W) had a mean of 27% proximal droplets in three ejaculates. This high incidence might be expected to affect fertility, although two out of three mares inseminated with the ejaculates analysed in this study became pregnant. The other stallions had 65–75% morphologically normal spermatozoa with a low incidence of individual abnormalities: fertility rates varied from 50 to 78%.

The chromatin quality of all the ejaculates used in this study would have been considered either excellent or good according to Love's classification scheme [10]. Love, furthermore, suggested that DFI levels above 27% were linked to reduced fertility [10].

It should be noted that the mean pregnancy rate used here is only a rough guide to the fertility of the ejaculates since it does not take into account such extraneous variables as female factors and inseminator differences. Furthermore, some mares were inseminated several times during each oestrous using different ejaculates from the same stallion, making it impossible to identify the source ejaculate of the fertilising spermatozoon. These factors are a recognized difficulty in reporting "fertility" in horses [23].

The amount of seminal plasma included in extended semen doses for AI is thought to influence DNA quality markedly, with detrimental effects becoming apparent when semen samples are cooled and stored [10]. Stallion ejaculates to be transported for artificial insemination are extended with a suitable medium, e.g. Kenney's extender, to give an insemination dose of one billion spermatozoa in 20 mL, and consequently there is variable "dilution" of the seminal plasma between different ejaculates. This differential dilution of seminal plasma and, consequently, the exposure of spermatozoa to its potentially chromatin-damaging effects, may help to explain why there is so much apparent variation in the fertility of stallion ejaculates. Preliminary observations carried out on five ejaculates, at the beginning of the study reported here, showed a highly variable DFI result when extended semen was stored, cooled, for 24 hours in transport boxes: for two ejaculates, values of DFI rose to > 25%, whereas the remaining three ejaculates showed much smaller increase in DFI of around 2–5%. It is intended to look at chromatin integrity during storage of sperm samples at different temperatures in a later experiment.

In conclusion, sperm morphology and sperm chromatin structure were shown to be inter-related and both parameters were, in turn, related to pregnancy rates. Either parameter could be useful as an indicator for predicting sub-fertility of individual stallions. However, both sperm morphology and SCSA should be assessed regularly, since these parameters may change from breeding season to breeding season, an effect which will be investigated in future studies.

## Authors' contributions

JMM participated in the design of the study, supervised and participated in data collection at Flyinge AB, was responsible for analysis and interpretation of the data and drafted the manuscript. AJ carried out the chromatin analysis. A-MD assisted in coordination of the study and supervised the morphology analysis. LH assisted in data collection. TS supervised semen collection. HR-M participated in the design and coordination of the study and helped to draft the manuscript. All authors have read and approved the final manuscript.
